# Sexually Transmitted Infection Co-testing in a Large Urban Emergency Department

**DOI:** 10.5811/westjem.18404

**Published:** 2024-04-09

**Authors:** James S. Ford, Joseph C. Morrison, Jenny L. Wagner, Disha Nangia, Stephanie Voong, Cynthia G. Matsumoto, Tasleem Chechi, Nam Tran, Larissa May

**Affiliations:** * University of California San Francisco, Department of Emergency Medicine, San Francisco, California; † University of California Davis, Davis, California; ‡ California State University, Department of Public Health, Sacramento, California; § University of California Davis, School of Medicine, Sacramento, California; ∥ University of California Davis Health, Department of Emergency Medicine, Sacramento, California; ¶ University of California Davis Health, Learning Health System, Department of Population Health and Accountable Care, Sacramento, California; # University of California Davis Health, Department of Pathology and Laboratory Medicine, Sacramento, California

**Keywords:** emergency department, sexually transmitted infection, sexually transmitted disease, public health, human immunodeficiency virus, HIV, hepatitis C virus, syphilis, gonorrhea, chlamydia

## Abstract

**Introduction:**

The incidence of sexually transmitted infections (STI) increased in the United States between 2017–2021. There is limited data describing STI co-testing practices and the prevalence of STI co-infections in emergency departments (ED). In this study, we aimed to describe the prevalence of co-testing and co-infection of HIV, hepatitis C virus (HCV), syphilis, gonorrhea, and chlamydia, in a large, academic ED.

**Methods:**

This was a single-center, retrospective cross-sectional study of ED patients tested for HIV, HCV, syphilis, gonorrhea or chlamydia between November 27, 2018–May 26, 2019. In 2018, the study institution implemented an ED-based infectious diseases screening program in which any patient being tested for gonorrhea/chlamydia was eligible for opt-out syphilis screening, and any patient 18–64 years who was having blood drawn for any clinical purpose was eligible for opt-out HIV and HCV screening. We analyzed data from all ED patients ≥13 years who had undergone STI testing. The outcomes of interest included prevalence of STI testing/co-testing and the prevalence of STI infection/co-infection. We describe data with simple descriptive statistics.

**Results:**

During the study period there were 30,767 ED encounters for patients ≥13 years (mean age: 43 ± 14 years, 52% female), and 7,866 (26%) were tested for at least one of HIV, HCV, syphilis, gonorrhea, or chlamydia. We observed the following testing frequencies (and prevalence of infection): HCV, 7,539 (5.0%); HIV, 7,359 (0.9%); gonorrhea, 574 (6.1%); chlamydia, 574 (9.8%); and syphilis, 420 (10.5%). Infectious etiologies with universal testing protocols (HIV and HCV) made up the majority of STI testing. In patients with syphilis, co-infection with chlamydia (21%, 9/44) and HIV (9%, 4/44) was high. In patients with gonorrhea, co-infection with chlamydia (23%, 8/35) and syphilis (9%, 3/35) was high, and in patients with chlamydia, co-infection with syphilis (16%, 9/56) and gonorrhea (14%, 8/56) was high. Patients with HCV had low co-infection proportions (<2%).

**Conclusion:**

Prevalence of STI co-testing was low among patients with clinical suspicion for STIs; however, co-infection prevalence was high in several co-infection pairings. Future efforts are needed to improve STI co-testing rates among high-risk individuals.

Population Health Research Capsule What do we already know about this issue?

*Data on sexually transmitted infection (STI) testing and prevalence are limited in the emergency department (ED) setting.*

What was the research question?

*What is the prevalence of STI testing, co-testing and co-infection among ED patients*.

What was the major quantitative finding of the study?

*Co-testing for STIs was infrequent, but co-infection with chlamydia was high among patients with syphilis (21%) and gonorrhea (23%).*

How does this improve population health?

*This study highlights the need to improve STI co-testing rates among high-risk individuals.*

## INTRODUCTION

An estimated one in five individuals in the United States (US) are infected with a sexually transmitted infection (STI).^1^^,^^2^ Between 2017–2021, the incidence of syphilis and gonorrhea increased and the incidence of chlamydia infections remained high.^2^ With widespread use of antiretroviral treatment, the overall incidence of HIV has declined over the same period, but incidence has plateaued in certain high-risk groups, such as people who inject drugs.^3^ While curative treatment for HCV became available in the US in 2011, the incidence of HCV doubled between 2013–2020.^4^^,^^5^ Moreover, just 33% of those with chronic HCV have been cured, and less than 17% of young (<40 years), uninsured patients have achieved sustained viral clearance.^6^ Low testing frequencies, patient unawareness of infection, poor access to traditional treatment settings (ie, primary care clinics) and re-infection following cure all contribute to these sub-optimal data.^6^^,^^7^

The emergency department (ED) is an important safety net for underserved, high-risk populations, making it a vital setting to deliver healthcare services to patients without access to primary care.^8^^,^^9^ Emergency department-based infectious diseases screening programs have demonstrated success in identifying STIs and linking patients to treatment.^10^^–^^12^ It is well known that contraction of one STI increases a patient’s risk of co-infection with other STIs.^13^^,^^14^ One ED-based study showed that among patients who received testing for STI, co-testing for a second STI was as low as 8%; however, this study did not report prevalence of infection/co-infection.^15^ Other ED-based studies report prevalence of co-infection but only single STI pairings.^10^^,^^15^^–^^17^ Understanding ED STI co-testing frequencies and prevalence of co-infections is imperative for optimizing public health infectious disease surveillance and treatment, particularly among patients without access to traditional primary care services. In this study, we aimed to describe co-testing and co-infection prevalence of HIV, hepatitis C virus (HCV), syphilis, gonorrhea, and chlamydia, in a large, academic ED.

## METHODS

### Overview

**Figure 1.**
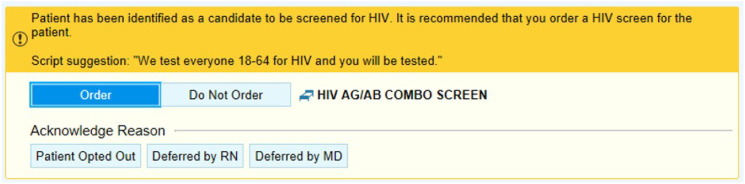
Example of a best practice alert inside the electronic health record.

In 2018, the study institution implemented an opt-out, ED-based infectious diseases screening program that employed electronic health record (EHR) best practice alerts (BPA). Any patient being tested for gonorrhea/chlamydia was eligible for opt-out syphilis screening. Additionally, any ED patient 18–64 years of age who was having blood drawn for any clinical purpose, was eligible for opt-out HIV and HCV screening. Funding for lab tests was obtained by charging the patient’s insurance, a billing strategy employed by similar screening programs and studies.^18^ If a patient requested that their insurance not be charged, or they did not have insurance, testing was paid for by the program grant. Physicians (including all residents), nurse practitioners and physician assistants could order testing. The full details of these screening programs have been previously described.^10^^–^^12^ An example BPA is available in Figure 1. In this study we examined STI testing/co-testing frequencies and infection prevalence in the ED. As data was initially collected for quality assurance purposes, the study was deemed not to be human subjects research by the Institutional Review Board Quality Improvement Self-Certification Tool.

### Study Design and Setting

This was a retrospective, cross-sectional study of ED patients tested for HIV, HCV, syphilis, gonorrhea, or chlamydia during the six-month period following implementation of the ED-based infectious diseases screening program. The study institution was a quaternary care, academic, Level I trauma center in Northern California that sees more than 80,000 patient visits annually.

### Selection of Participants

We included data from all patients ≥13 years who had undergone testing for one or more of HIV, HCV, syphilis, gonorrhea, or chlamydia in the ED between November 27, 2018–May 26, 2019.

### Measurements

We abtracted data from the EHR (Epic Systems Corp, Verona, WI) using computer-generated reports by querying patients who had received ED STI tests during the study period. We included demographic factors (age, gender, race, ethnicity) and results of STI testing. The data analyst responsible for procuring these reports was blinded to the hypothesis of the study. We defined STI co-testing as testing for two or more of the following STIs: gonorrhea, chlamydia, syphilis, HIV, and HCV. To prevent duplicate data, we included only a patient’s first ED visit where they received HCV testing when calculating co-testing/co-infection prevalence. We examined subsequent testing that occurred in future ED visits to identify instances where broader STI testing could have identified infections earlier. Data was stored in de-identified datasets, and patients were given unique identifiers to maintain patient confidentiality.

HIV screening was performed using a HIV P24 antigen (Ag) and HIV-1/HIV-2 antibody (Ab) combination test with the ARCHITECT i1000SR immunoanalyzer (Abbott Laboratories, Abbott Park, IL), and diagnoses were confirmed using Bio-Rad Rapid Test Multispot HIV-1/HIV-2 Ab reflex testing (Bio-Rad Laboratories, Inc, Hercules, CA). Screening for HCV in the ED was performed using a chemiluminescent anti-HCV ARCHITECT i1000SR immunoassay, and diagnoses were confirmed by HCV ribonucleic acid viral load (VL) with Cobas HCV 4800 assay (Roche Molecular Systems, Pleasanton, CA). Patients were considered positive for HCV only if they had a detectable VL. Multiplex gonorrhea and chlamydia urine polymerase chain reaction testing was also performed via the Cobas 4800 assay. Patients were tested for *Treponema pallidum* IgM/IgG antibody (TPA) using Bio-Rad’s multiplex flow immunoassay (MFI), Bioplex 2200.^19^ Specimens with reactive TPA MFI results underwent reflexive confirmatory quantitative non-treponemal assay testing, using rapid plasma reagin. If test results were discordant, the specimen was tested reflexively using the *T pallidum* particle agglutination test as an additional confirmatory treponemal test.

### Outcomes

The outcomes of interest included the prevalence of STI testing/co-testing and prevalence of STI infection/co-infection.

### Analysis

We described data using descriptive statistics. Categorical variables were expressed as percentages and proportions, and continuous variables were expressed as means ± standard deviations. We performed all statistical analyses using Stata 15.1 (StataCorp LLC, College Station, TX).

## RESULTS

### Patient Characteristics

There were 30,767 ED patient encounters for patients aged ≥13 years during the study period. Of these, 7,866 (26%) were tested for at least one of HIV, HCV, syphilis, gonorrhea, or chlamydia. The mean age of patients was 43 ± 14 years, and 4,077 (52%) were female. The most common race was White (39%), and most patients were non-Hispanic (76%). Most patients tested had Medicaid insurance (56%). See Table 1 for full patient characteristics.

**Table 1.** Characteristics of 7,866 emergency department patients who underwent testing for at least one sexually transmitted infection.

Characteristic	Value
Mean age (years)^1^	43 ± 14
Genderr	
Male	48% (3,789)
Female	52% (4,077)
Race/ethnicity^2^	
White	39% (3,034)
Black	22% (1,698)
Asian	7% (517)
Mixed/other	32% (2,517)
Ethnicity^3^	
Hispanic	24% (1,845)
Non-Hispanic	76% (5,933)
Sexuality (self-identified)^4^	
Heterosexual	93% (1,386)
LGBTQ	7% (105)
Housing status^5^	
Domiciled	91% (5,982)
Undomiciled	9% (614)
Insurance type	
Private	27% (2,162)
Medicare	13% (1,025)
Medicaid	56% (4,399)
Self/uninsured	4% (280)

^1^ Reported as mean ± standard deviation.

^2^ Data missing for 100 patients.

^3^ Data missing for 88 patients.

^4^ Data missing for 6,375 patients.

^5^ Data missing for 1,270 patients.

*LGBTQ*, Lesbian, Gay, Bisexual, Transgender, Queer.

### Prevalence of Sexually Transmitted Infection Testing/Co-Testing

The most commonly tested STIs were those with universal screening indications: HCV (24.5%, 7,539/30,767); and HIV (23.9%, 7,359/30,767). Gonorrhea/chlamydia (1.9%, 74) testing was more common than syphilis testing (1.4%, 420/30,767). Of those who received testing for STIs, 6.5% (508/7,866) were tested for a single STI. Patients were tested for two or more STIs in 95.6% (7,521/7,866) of cases and three or more STIs in 5.6% (437/7,866) of cases. Patients were tested for HIV, HCV, syphilis, and gonorrhea/chlamydia in 3.6% (286/7,866) of cases. See Table 2 for overall co-testing.

**Table 2.** Overall testing/co-testing proportions among emergency department (ED) patients tested for sexually transmitted infections during their first ED visit.

One STI tested	Testing proportion
HCV only	24.5% (7,539/30,767)
HIV only	23.9% (7,354/30,767)
Syphilis only	1.4% (420/30,767)
Gonorrhea	1.9% (574/30,676)
Chlamydia	1.9% (574/30,676)
**Two STIs co-tested**	**Co-testing proportion**
Gonorrhea + Chlamydia^1^	100% (574/574)
HCV + HIV	95% (7,240/7,650)
HCV + syphilis	4.4% (333/7,626)
HCV + Gonorrhea	4.4% (344/7,769)
HCV + Chlamydia	4.4% (344/7,769)
HIV + syphilis	4.5% (357/7,417)
HIV + Gonorrhea	4.6% (346/7,582)
HIV + Chlamydia	4.6% (346/7,582)
Syphilis + Gonorrhea	57% (361/633)
Syphilis + Chlamydia	57% (361/633)
**Three STIs co-tested**	**Co-testing proportion**
HCV + HIV + syphilis	2.1% (314/14,680)
HCV + HIV + Gonorrhea	2.2% (319/14,829)
HCV + HIV + Chlamydia	2.2% (319/14,829)
HCV + syphilis + Gonorrhea	2.0% (303/14,861)
HCV + syphilis + Chlamydia	2.0% (303/14,861)
HIV + syphilis + Gonorrhea	4.0% (306/7,736)
HIV + syphilis + Chlamydia	4.0% (306/7,736)
**Four STIs co-tested**	**Co-testing proportion**
HCV + HIV + Syphilis + Gonorrhea	286 (3.6%)
HCV + HIV + Syphilis + Chlamydia	286 (3.6%)
**All five STIs co-tested**	**Co-testing proportion**
HCV + HIV + syphilis + Gonorrhea + Chlamydia	286 (3.6%)

^1^ Gonorrhea and chlamydia were always tested together.

*STI*, sexually transmitted infection; *HCV*, hepatitis C virus.

### Prevalence of Infection/Co-Infection

The seroprevalence of infection was highest for syphilis (44/420, 10.5% [95% CI 7.7–13.8]), followed by chlamydia (56/574, 9.8% [95% CI 7.4–12.5]), gonorrhea (35/574, 6.1% [95% CI 4.3–8.4]), HCV (373/7,470, 5.0% [95% CI 4.5–5.5]), and HIV (67/7,354, 0.9% [95% CI 0.7–1.2]). Among 67 patients who tested positive for HIV, HCV was the most common co-infection (seven patients, 10.4%). Among 373 patients who tested positive for HCV, HIV was the most common co-infection (seven, 0.9%). Among 44 patients who tested positive for syphilis, chlamydia was the most common co-infection (nine, 20.5%). Among 35 patients who tested positive for gonorrhea, chlamydia was the most common co-infection (eight, 22.9%). Among patients who tested positive for chlamydia, syphilis was the most common co-infection (nine, 16.1%). One patient was infected with three STIs (HCV, HIV, and syphilis). No patients were infected with more than three concurrent STIs. Overall co-infection data is available in Table 3.

**Table 3.** Infection and co-infection proportions for sexually transmitted infections.

Infection type	% (Proportion)
Syphilis infection	10.5% (44/420)
Chlamydia co-infection	20.5% (9/44)
HIV co-infection	9.1% (4/44)
Gonorrhea co-infection	6.8% (3/44)
HCV co-infection	2.3% (1/44)
Gonorrhea infection	6.1% (35/574)
Chlamydia co-infection	22.9% (8/35)
Syphilis co-infection	8.6% (3/35)
HIV co-infection	2.9% (1/35)
HCV co-infection	2.9% (1/35)
Chlamydia infection	9.8% (56/574)
Syphilis co-infection	16.1% (9/56)
Gonorrhea co-infection	14.3% (8/56)
HCV co-infection	1.8% (1/56)
HIV co-infection	0%
HIV infection	0.9% (67/7,354)
HCV co-infection	10.4% (7/67)
Syphilis co-infection	5.9% (4/67)
Gonorrhea co-infection	1.5 (1/67)
Chlamydia co-infection	0%
HCV infection	5.0% (373/7,470)
HIV co-infection	1.9% (7/373)
Chlamydia co-infection	0.3% (1/373)
Gonorrhea co-infection	0.3 % (1/373)
Syphilis co-infection	0.3% (1/373)

Gonorrhea and chlamydia were always tested together.

*HCV*, hepatitis C virus.

### Potentially Missed Diagnoses

A total of 633 patients received targeted STI testing due to clinical concern during their first ED visit (tested for combo gonorrhea/chlamydia and/or syphilis). However, co-testing between syphilis and gonorrhea/ chlamydia occurred in only 57% (361/633) of these testing encounters. Only 63% (397/633) of these patients received HIV co-testing, and only 59% received HCV co-testing. Some patients received STI testing for one or more STIs, but not all five STIs (gonorrhea, chlamydia, syphilis, HIV, HCV), during their first ED visit. In this group with incomplete STI testing, we assessed whether patients received further STI testing in any of their next four documented ED visits within the study period and found the following testing counts and positive results: HCV, 81 (9 positive [11.1%]); HIV, 61 (1 positive [1.6%]); syphilis, 49 (3 positive [6.1%]); and gonorrhea/chlamydia, 55 (1 gonorrhea positive [1.8%]).

## DISCUSSION

In our study we examined STI testing/co-testing and infection/co-infection prevalence in ED patients who were tested for at least one of HIV, HCV, syphilis, gonorrhea, or chlamydia. To our knowledge, this is the first ED-based study to report ED STI co-testing and co-infection frequencies for every combination of gonorrhea, chlamydia, syphilis, HIV, and HCV. Overall, STI co-testing was infrequent, but co-infection prevalence was high among several STI co-test pairings.

The HIV/HCV testing co-testing occurred frequently, likely related to the presence of the universal screening BPA. According to the US Centers for Disease Control and Prevention, approximately 21% of individuals with HIV in high-risk populations (ie, men who have sex with men, people who use drugs) are co-infected with HCV.^20^ While published data is limited, previous ED-based studies found that 8–33% patients with HIV were co-infected with HCV.^21^^–^^24^ In our study, 10.5% of patients with HIV were co-infected with HCV, but only 1.9% of patients with HCV were co-infected with HIV. Previous studies in this ED population found that patients shared some (male gender, unhoused status, history of illicit drug use, and Medicare insurance status) but not all risk factors for infection.^25^^,^^26^ It is possible that co-infection proportions may differ among patients with HCV and HIV due to some other unmeasured risk factor. Alternatively, given the immunosuppressive properties of HIV, patients who are exposed to HCV may be more likely to progress to a chronic infection.^27^

Among patients with gonorrhea, 23% were co-infected with chlamydia. Conversely, only 14% of those infected with chlamydia were co-infected with gonorrhea. This differential co-infection pattern has been previously reported in at least one other ED-based study (gonorrhea+, chlamydia+: 44%; chlamydia+, gonorrhea+: 17%).^28^ Among patients with syphilis, we also observed a high prevalence of chlamydia co-infection (21%). In our study, there was a BPA that prompted clinicians to test patients for syphilis who were undergoing gonorrhea/chlamydia testing, and co-testing occurred in just 57% of patients. Previous studies report the prevalence of syphilis and gonorrhea/chlamydia co-testing (9–39%); however, we could not find any ED-based studies that reported proportions of co-infection.^29^ Similarly, we found that patients who were tested for syphilis and/or gonorrhea/chlamydia, co-testing for HIV and HCV occurred infrequently. Given that patients with syphilis, gonorrhea, and chlamydia had the highest prevalence of co-infection with other STIs. These instances represent potential missed opportunities for diagnosis and linkage to care.

In our study, there were several patients who tested positive for specific STIs in subsequent ED visits and were not tested for these STIs in their initial visit. It is possible that patients contracted the STI exposure after the index ED visit, and had they been tested at the index visit they may have been negative. However, it is also possible that these diagnoses were present at the index visit and were missed, suggesting that increased co-testing can lead to increased diagnosis of clinically significant co-infections with the potential to reduce transmission in the community.

## LIMITATIONS

This was a retrospective study with data obtained from the EHR at a single institution; thus, our findings may not be generalizable to all settings. Our study had multiple BPAs in place that likely influenced clinician co-testing behavior. We did not report chief complaints, making it difficult to differentiate patients with true clinical indication for testing, and patients who were being screened as part of a screening protocol. Since testing for syphilis and gonorrhea/chlamydia was not universal, the reported proportions are unlikely to represent the prevalence for the whole ED, but rather the prevalence of infection among patients with clinical suspicion for STI. While HIV and HCV screening was universally ordered for most patients undergoing bloodwork, patients could still opt out, which may have biased the prevalence estimates for these infections.

## CONCLUSION

Prevalence of co-testing for sexually transmitted infection was low among patients with clinical suspicion for STI; however, co-infection prevalence was high in several co-infection pairings. Encounters with single STI testing represent a missed opportunity to screen for co-infections. Future efforts are needed to improve STI co-testing rates among high-risk individuals. With the incidence of many STIs increasing, the ED can serve as an important screening setting for STIs, especially in patients without access to traditional outpatient services.
